# Polyanhydride Nanoparticle Delivery Platform Dramatically Enhances Killing of Filarial Worms

**DOI:** 10.1371/journal.pntd.0004173

**Published:** 2015-10-23

**Authors:** Andrea M. Binnebose, Shannon L. Haughney, Richard Martin, Paula M. Imerman, Balaji Narasimhan, Bryan H. Bellaire

**Affiliations:** 1 Department of Veterinary Microbiology and Preventive Medicine, Iowa State University, Ames, Iowa, United States of America; 2 Department of Chemical and Biological Engineering, Iowa State University, Ames, Iowa, United States of America; 3 Department of Biomedical Sciences, Iowa State University, Ames, Iowa, United States of America; 4 Veterinary Diagnostic Laboratory, Iowa State University, Ames, Iowa, United States of America; University of Liverpool, UNITED KINGDOM

## Abstract

Filarial diseases represent a significant social and economic burden to over 120 million people worldwide and are caused by endoparasites that require the presence of symbiotic bacteria of the genus *Wolbachia* for fertility and viability of the host parasite. Targeting *Wolbachia* for elimination is a therapeutic approach that shows promise in the treatment of onchocerciasis and lymphatic filariasis. Here we demonstrate the use of a biodegradable polyanhydride nanoparticle-based platform for the co-delivery of the antibiotic doxycycline with the antiparasitic drug, ivermectin, to reduce microfilarial burden and rapidly kill adult worms. When doxycycline and ivermectin were co-delivered within polyanhydride nanoparticles, effective killing of adult female *Brugia malayi* filarial worms was achieved with approximately 4,000-fold reduction in the amount of drug used. Additionally the time to death of the macrofilaria was also significantly reduced (five-fold) when the anti-filarial drug cocktail was delivered within polyanhydride nanoparticles. We hypothesize that the mechanism behind this dramatically enhanced killing of the macrofilaria is the ability of the polyanhydride nanoparticles to behave as a Trojan horse and penetrate the cuticle, bypassing excretory pumps of *B*. *malayi*, and effectively deliver drug directly to both the worm and *Wolbachia* at high enough microenvironmental concentrations to cause death. These provocative findings may have significant consequences for the reduction in the amount of drug and the length of treatment required for filarial infections in terms of patient compliance and reduced cost of treatment.

## Author Summary

Infection with the filarial endoparasites *Brugia malayi* and its symbiotic bacteria *Wolbachia* represent a significant burden to both humans and animals. Current treatment protocols include use of multiple drugs over a course of months to years, resulting in high costs, undesirable side effects, and poor patient compliance. By encapsulating two of these drugs, ivermectin and doxycycline, into biodegradable polyanhydride nanoparticles, we report the ability to effectively kill adult *B*. *malayi* with up to a 4,000-fold reduction in the amount of drug used. These results demonstrate a promising role for the use of nanoscale drug carriers to reduce both the course of treatment and the amount of drug needed to increase affordability of lymphatic filariasis treatment and enhance patient compliance.

## Introduction

Filarial parasites belonging to the family Onchocercidae remain a significant global burden, endemic in over 80 countries worldwide, particularly India and sub-Saharan Africa [1], and infecting up to 120 million individuals [2]. The major human diseases caused by filaria are Lymphatic Filariasis (LF), River Blindness (RB), and Loiasis. Of the Onchocercidae, *Onchocerca volvulus* causes RB while LF results from the infections of *Wuchereria bancrofti* and *Brugia malayi*. Infection is initiated through arthropod transmission of larvae into the skin of a vertebrate host where they feed, mature into fertile adults and reproduce. Adult worms survive within these tissues for several years shedding thousands of microfilariae (MF) that migrate through the skin and lymphatics and distribute throughout the body. Morbidity associated with infection stems from both the persistence of the worm in the skin and the lymphatics that creates scarring and fluid accumulation and the MF causing debilitating chronic dermatitis due to irritation of MF antigen [3]. Resident within *W*. *bancrofti* and *B*. *malayi* parasites at all stages of life are the endosymbiotic bacteria, *Wolbachia*. These obligate intracellular bacteria are members of the Rickettsialaes order of α-proteobacteria [4] that also contain the mammalian pathogens in the genera *Rickettsia*, *Brucella*, *Bartonella*, *Ehrlichia* and *Anaplasma* [5]. Typically, *Wolbachia* are present within hypodermal cells of the lateral cords of both sexes, however, vertical transmission of the bacteria to MF is facilitated by the presence of bacteria in the ovaries, oocytes and embryos during development in females [6].

Within the last decade increasing concerns have arisen regarding the ability to effectively control and eradicate current infections of the filarial endoparasitic worms that cause onchocerciasis and LF [7]. It is estimated that 120 million people are currently suffering from LF and more than one billion individuals in 73 countries are at risk of developing LF [8]. In 2000, the WHO initiated a call to eliminate LF as a public-health problem by 2020 [9]. Significant steps have been taken towards this eradication effort but have been mainly limited to the use of mass drug administration of a microfilaricide to interrupt transmission and diminish morbidity. This approach has reduced the reoccurrence in some countries by 46% over the last 13 years [2,9]. Drugs with the greatest efficacy toward LF have been limited to trials of annual, bi-annual, single-dose, multiple dose, and combinations of either ivermectin or diethylcarbamazine with albendazole [7,10]. Recently, antimicrobial treatments such as doxycycline have been added to antifilarial regimens to target the symbiotic intracellular bacteria *Wolbachia* [11,12,13]. Anti-*Wolbachia* drugs have been shown to reduce the pathogenicity and reproductive capacity of adult filarial worms [14]. The limitations associated with the use of the above drugs are four-fold: specific restrictions on patient age and health status due to cytotoxicity; the inability to reach the deep tissues where the adult filarial nematodes reside; counter-indications in areas endemic for the *Loa loa* parasite; and poor patient compliance [15]. These limitations have created an urgent need to seek new technologies that can be used to dramatically improve therapy protocols.

In the parasite, *Wolbachia* are vertically transferred from the ovaries of the adult into embryos that gestate and are shed as MF into the surrounding tissue [16]. In the adult female, the bacteria contribute to reproduction and embryo development. Treating adult worms *in vitro* and *in vivo* with tetracyclines, including doxycycline, to eliminate *Wolbachia* has been shown to halt embryogenesis, reduce the amount and viability of MF shed [14,17,18] in tissues, and lead to death of the adult worm in 6 to 12 months [12,19]. Loss of viability in the adult worm through anti-*Wolbachia* therapy is precipitated by loss of the endosymbiont, triggering apoptosis and tissue disruption [20]. Another antibiotic that is effective against *Rickettsiae* is rifampicin, which is better tolerated in children and pregnant patients, is able to kill *Wolbachia* in *Onchocerca* parasites *in vitro* [21]. However, rifampicin is less effective than doxycycline *in vivo* [18,22]. Additional therapeutic options for doxycycline are to combine it with other current antiparasitic drugs; however combination therapies present difficulties for drugs that have different half-lives, for widespread drug administration programs that suffer from poor patient compliance, and for long treatment regimens that require multiple years of therapy [23].

Amphiphilic polyanhydride nanoparticles have been studied extensively as carriers for drugs and vaccines [24,25,26,27,28]. These biodegradable materials degrade into dicarboxylic acids upon scission of the anhydride bond, rendering them highly biocompatible. These materials are generally hydrophobic and this confers a surface erosion mechanism to devices made of polyanhydrides [29,30]. The advantage of such a surface erosion mechanism is that payloads can be released in a sustained and predictable manner, providing a so-called zero order release profile, in which the release rate of the payload is constant. Such release profiles enable single dose therapies, maximize the time over which the *in vivo* drug concentration is maintained within the therapeutic window, and enhance patient compliance. This nanoscale platform also has the capability to be a delivery system for combination therapies that carry payloads to hard to reach tissues, thereby offering the potential to increase the efficacy of the drug-nematode interaction. The ability of the polyanhydride nanoparticles to slowly erode and release the cargo molecule in a controlled manner can also allow for specificity against both the adult nematode and *Wolbachia* [11]. In this work, we demonstrate that co-delivery of doxycycline and ivermectin through encapsulation into polyanhydride nanoparticles effectively kills adult *B*. *malayi* filarial worms 8-fold faster with up to a 4,000-fold reduction in the amount of drug used. We hypothesize that the mechanism behind this enhanced killing of the macrofilaria is the ability of the nanoparticles to penetrate the outer membrane of the *B*. *malayi* worm and effectively deliver drug directly to the worm and its symbiotic bacteria *Wolbachia* at high enough microenvironment concentrations to cause death. The approach described herein holds great promise to interrupt the life cycle of the nematode not only by reducing the number of MF, but also by improving macrofilaricidal activity. These findings have the potential to dramatically enhance the treatment of patients suffering from debilitating filarial diseases such as LF and RB.

## Materials and Methods

### Acquisition and storage of *B*. *malayi* and microfilariae

Live adult *B*. *malayi* females, males, and microfilariae were acquired from the NIH/NIAID Infectious Disease Filariasis Research Reagent Repository Center (FR3) at the University of Georgia (Athens, GA). Adult worms were maintained in non-phenol red Roswell Park Memorial Institute (RPMI) 1640 medium supplemented with 10% heat-inactivated fetal bovine serum (FBS) and 1% penicillin-streptomycin. The *B*. *malayi* were held in an incubator at a temperature of 37°C supplemented with 5% carbon dioxide (CO_2_). Female and male worms were stored individually in 48 well microtiter plates containing 1 mL of RPMI-1640. Previously shed microfilariae were housed in 50 mL conical tubes containing 25 mL of RPMI-1640. Upon arrival of the filarial worms they were separated and placed into individual 48 well microtiter plates that contained the previously described media.

### Motility scoring

The motility of the adult worms treated with various formulations was observed for 30 seconds by utilizing a 2X objective on a Nikon Microscope and scored utilizing the following 0–5 scoring system: zero percent motility reduction, head and tail uninhibited as well as mid-section unaffected = **5**; 1 to 25% motility reduction, ability to visualize both the head and tail movements easily as well as midsection = **4**; 26 to 49% motility reduction, showing a partial mid-section paralysis = **3**; 50 to 74% motility reduction, showing full mid-section paralysis followed by a substantial reduced movement in the head and tail = **2**; 75 to 99% motility reduction, showing full mid-section paralysis followed by either head and or tail paralysis but not limited to occasional movement over a 30 second time period = **1**; and a score of **0** represented 100% death (i.e., non-motile). The loss of motility of the adult worms was compared with that of respective untreated controls.

### Synthesis of ivermectin and doxycycline-loaded polyanhydride nanoparticles

#### Materials

For the synthesis of the 1,6-bis(*p*-carboxyphenoxy)hexane (CPH) and 1,8-bis(*p*-carboxyphenoxy)-3,6-dioxaoctane (CPTEG) monomers and the corresponding 20:80 CPTEG:CPH copolymer, and the ivermectin and doxycycline-loaded nanoparticles, acetic acid, acetic anhydride, acetone, acetonitrile, chloroform, dimethyl formamide, ethyl ether, hexane, methylene chloride, pentane, petroleum ether, potassium carbonate, sodium hydroxide, sulfuric acid, and toluene were purchased from Fisher Scientific (Fairlawn, NJ). The chemicals 1,6-dibromohexane, 1-methyl-2-pyrrolidinone, hydroxybenzoic acid, N,N-dimethylacetamide, sebacic acid, and tri-ethylene glycol were obtained from Sigma Aldrich (St. Louis, MO). The chemical 4-*p*-fluorobenzonitrile was purchased from Apollo Scientific (Cheshire, UK). For ^1^H NMR analysis deuterated chloroform and deuterated dimethyl sulfoxide were purchased from Cambridge Isotope Laboratories (Andover, MA). Rhodamine B, a fluorescent dye, and the drugs, ivermectin and doxycycline, were purchased from Sigma Life Science (St. Louis, MO).

#### Polymer synthesis

Synthesis of the CPH and CPTEG monomers was performed as described elsewhere [34]. Copolymers containing 20:80 molar ratios of CPTEG and CPH were synthesized through melt condensation, as previously described [28,29]. Molecular weight and copolymer composition were confirmed using ^1^H NMR.

#### Nanoparticle synthesis

Doxycycline- and ivermectin-loaded 20:80 CPTEG:CPH nanoparticles were synthesized using solid/oil/oil nanoprecipitation, as previously described [25,31,32]. Briefly, ivermectin and doxycycline, each at 5% (w/w), and rhodamine B at 2% (w/w) of the total weight of the polymer were dissolved in methylene chloride at a concentration of 20 mg/mL followed by rapid precipitation into the anti-solvent, pentane. The polyanhydride nanoparticles were then collected using vacuum filtration. The drug-loaded particles were characterized for size and morphology by scanning electron microscopy (SEM, FEI Quanta SEM, Hillsboro, OR). So, every 100 mg of the final nanoparticle preparation would contain 5 mg of IVM, 5 mg of doxycycline and 2 mg of rhodamine.

### Drug release kinetics

The in vitro release kinetics of ivermectin and doxycycline from 20:80 CPTEG:CPH nanoparticles was determined using high performance liquid chromatography (HPLC). Nanoparticles were suspended in 1 mL of phosphate buffered saline (PBS, pH 7.2), followed by thorough sonication to suspend the particles and incubated at 37°C under constant shaking. At each time point, the tubes were centrifuged at 10,000 rcf for 10 min. The supernatant was removed and replaced with fresh PBS and returned to incubation.

The ivermectin was separated using a Zorbax C8 4.6 x 250 cm chromatography column using a mobile phase of tetrahydrofuran, acetonitrile, and water in a 40:40:20 volume ratio, respectively. The flow rate was set at 1 mL/min and ivermectin eluted at a retention time of 5.3 min. The ivermectin was quantified with fluorescent detection using an excitation wavelength of 365 nm and an emission wavelength of 475 nm with a gain of 10 and attenuation of 64 [33]. The doxycycline was quantified using a Varian Pursuit XRs 3 C18 150 x 2 mm column. The mobile phase consisted of water with 0.1% formic acid and acetonitrile at a 98:2 volume ratio and at a flow rate of 0.2 mL/min. After one minute the mobile phase was ramped to 100% acetonitrile over 4 min. Doxycycline was quantified with tandem mass spectroscopy (MS/MS) using the following conditions: Q1 to Q3 transitions of 445 to 154 at 30 V collision energy and 445 to 427.9 at 16 V collision energy [34]. The encapsulation efficiency of the particles was determined as described previously [35] and found to be approx. 100% for both drugs and for the rhodamine.

### Drug treatments of *B*. *malayi*

Ivermectin (22,23-dihydroavermectin B1) and doxycycline hyclate (C_22_H_24_N_2_O_8_ · HCl · 0.5H_2_O · 0.5C_2_H_6_O) were dissolved in DMSO at a final concentration of 0.02% v/v. RPMI-1640 medium was prepared such that the final concentrations of each drug in nanoparticles were 195, 49, 10, 5, 1.95, 0.049, 0.01, 0.005, and 0.001 μM, respectively. Control medium contained 0.02% DMSO and rhodamine B, but no drug. Individual worms that were previously placed into 48 well flat bottom culture plates upon arrival had new fresh media that contained 1 mL of RPMI-1640, 0.01% streptomycin and penicillin and 10% heat-inactivated fetal calf serum. *B*. *malayi* were incubated at 37° C and 5% CO_2_ for 1–2 hours for acclimatization to evaluate base line motility before the experiment began. The nanoparticle-encapsulated drugs and standard antifilarials (i.e., free drug with no nanoparticle encapsulation) were added as previously described above. For example, 2 mg of the 195 μM drug concentration nanoparticles were added to each well and correspondingly lower amounts of nanoparticles were added as the drug concentrations decreased. Controls received equal amounts of media but lacked the nanoparticles and standard antifilarials. *B*. *malayi* were incubated at 37°C and 5% CO_2_ and monitored for 14 days post treatment.

### Viability assessment

Reversibility assays where conducted 24 h following a 9 day course of treatment and or after the final individual within the soluble group reached a motility score of 0. Individual worms where transferred to a new 48 well microtiter plate that contained 1 mL of the previously described media and were placed into an incubator at a temperature of 37°C supplemented with 5% carbon dioxide (CO_2_). After 24 h a motility score was recorded. Upon completion of reversibility adult female worm viability was assessed utilizing a standard 3-(4,5-dimethylthiazol-2-yl)-2,5-diphenyltetrazolium bromide) (MTT)-formazan colorimetric assay. The same female worms used in the motility assay were gently blotted and transferred to a 96 well microtiter plate that contained 0.2 mL of 1mg/mL MTT in phosphate-buffered saline (pH 7.2) and incubated for 2 h at 37°C. The formazan formed was extracted in 0.15 mL of DMSO for 4 h at 23°C and the absorbance was measured at 570 nm using a spectrophotometer (FLUOstar Omega version 1.01). The mean absorbance of the formazan from the treated worms was compared with that of the controls. The viability of the treated worms was assessed by calculating the percent inhibition in motility and MTT reduction over the DMSO-treated control worms.

### MF shedding and motility

The effect of treatments on MF shedding and subsequent motility was assessed using two experimental approaches. The direct effect on MF was performed by collecting MF from the spent media of healthy untreated female worms. Spent media was centrifuged for 10 min at 200 rcf, the MF were re-suspended in fresh media and enumerated by counting using a 14.80 mm² grid plate. Aliquots containing at least 250 MF were dispensed into wells of a 96 well plate and treated in triplicate with either soluble drug, blank nanoparticles or nanoparticles containing drugs as indicated. Wells were then monitored and scored daily for MF movement. Motility of MF scoring was carried out using a 10X Nikon microscope and individual scores from five separate fields of view (FOV) were averaged to yield a motility score for each well. Scoring was adapted from a previously reported method by observing the movements of the anterior and posterior body over one min [36]. Data were analyzed by ANOVA and graphs were assembled using GraphPad Prism (v6.05). In separate experiments, the effect of treating adult female *Brugia* and their ability to release MF was monitored and recorded for 14 days. The motility of the shed MF was also recorded. Each experiment, the various treatment groups, and the different doses of the drugs were repeated for a minimum of three times.

### Nanoparticle uptake by *B*. *malayi*

To understand the interaction between the nanoparticles and the filarial worms, recently deceased adult worms (motility score of “0”) from treated and untreated experimental groups were fixed and prepared for confocal microscopy. Worms were removed from treatment wells, washed twice in pH 7.4 PBS and fixed with 4% paraformaldehyde for 1 h at room temperature. Worms were removed, rinsed with fresh PBS and mounted carefully onto glass slides using Prolong Gold with DAPI (Life Technologies). Slides were then stored in the dark at 4°C prior to imaging. Imaging was performed using an Olympus IX-71 laser-scanning confocal microscope (Fluoview 2000, v2.0) equipped with 405 nm, 488 nm, 559 nm and 635 nm lasers, spectral photomultiplier detection and a piezo-controlled stage. Laser power and exposure settings were set according to the negative (worms with no rhodamine) and positive (rhodamine containing nanoparticles) and maintained constant over all the experiments. Image stacks were collected simultaneously for bright field (transmitted light—grey scale), cell nuclei (DAPI—blue) and nanoparticles (rhodamine—red). Bright field and cell nuclei were used to identify various tissues, organs and structures in the worm such as oral opening, pharynx, uterus, the nerve ring, extracellular secretory apparatus, esophageal track and anal pore. Rendering of Z-stack data in three dimensions and minimal image processing was performed as described previously by our laboratory [37] using ImageJ v 1.49a (NIH). Software parameters for image processing remained constant for all image/data sets presented.

## Results

### Encapsulation of doxycycline and ivermectin into polyanhydride nanoparticles provides sustained drug release

**Fig 1:**
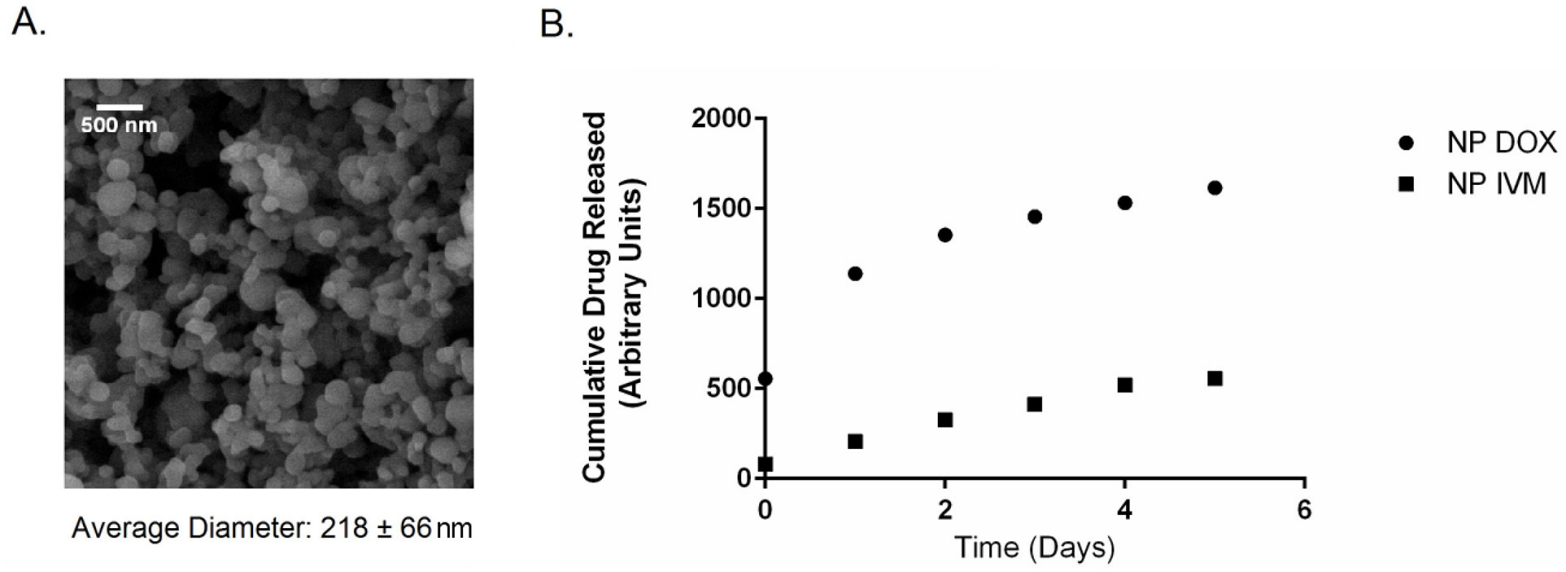
Panel A: Scanning electron photomicrograph of 20:80 CPTEG:CPH nanoparticles loaded with 5% IVM, 5% DOX, and 2% rhodamine. Scale bar: 500 nm. Panel B: Release kinetics of ivermectin (IVM) and doxycycline (DOX) from 20:80 CPTEG:CPH nanoparticles quantified using HPLC.

After encapsulation of the anti-filarial drugs by nanoprecipitation, the particle morphology and size were examined using scanning electron microscopy. Surface morphology was found to be consistent with previous work and the scanning electron photomicrographs are shown in Fig 1 (panel A) [26,27,38]. Additionally, particle size was also found to be consistent with previous work [37,39]. The mean diameter of the drug-containing 20:80 CPTEG:CPH nanoparticles was 218 ± 56 nm.

Using a HPLC assay as described in the Methods section, the release kinetics of ivermectin and doxycycline from the nanoparticles was quantified, as shown in Fig 1 (panel B). The 20:80 CPTEG:CPH nanoparticle formulation provided sustained release of both drugs over one week. That data showed that the doxycycline release profile from the hydrophobic 20:80 CPTEG:CPH nanoparticles showed a small burst effect, which is consistent with previous work [40]. It was also observed that there was a much larger burst release of doxycycline as compared to that of ivermectin.

### Polyanhydride nanoparticle-based drug delivery significantly increased *B*. *malayi* macrofilaria mortality and significantly reduced microfilarial shedding

**Fig 2:**
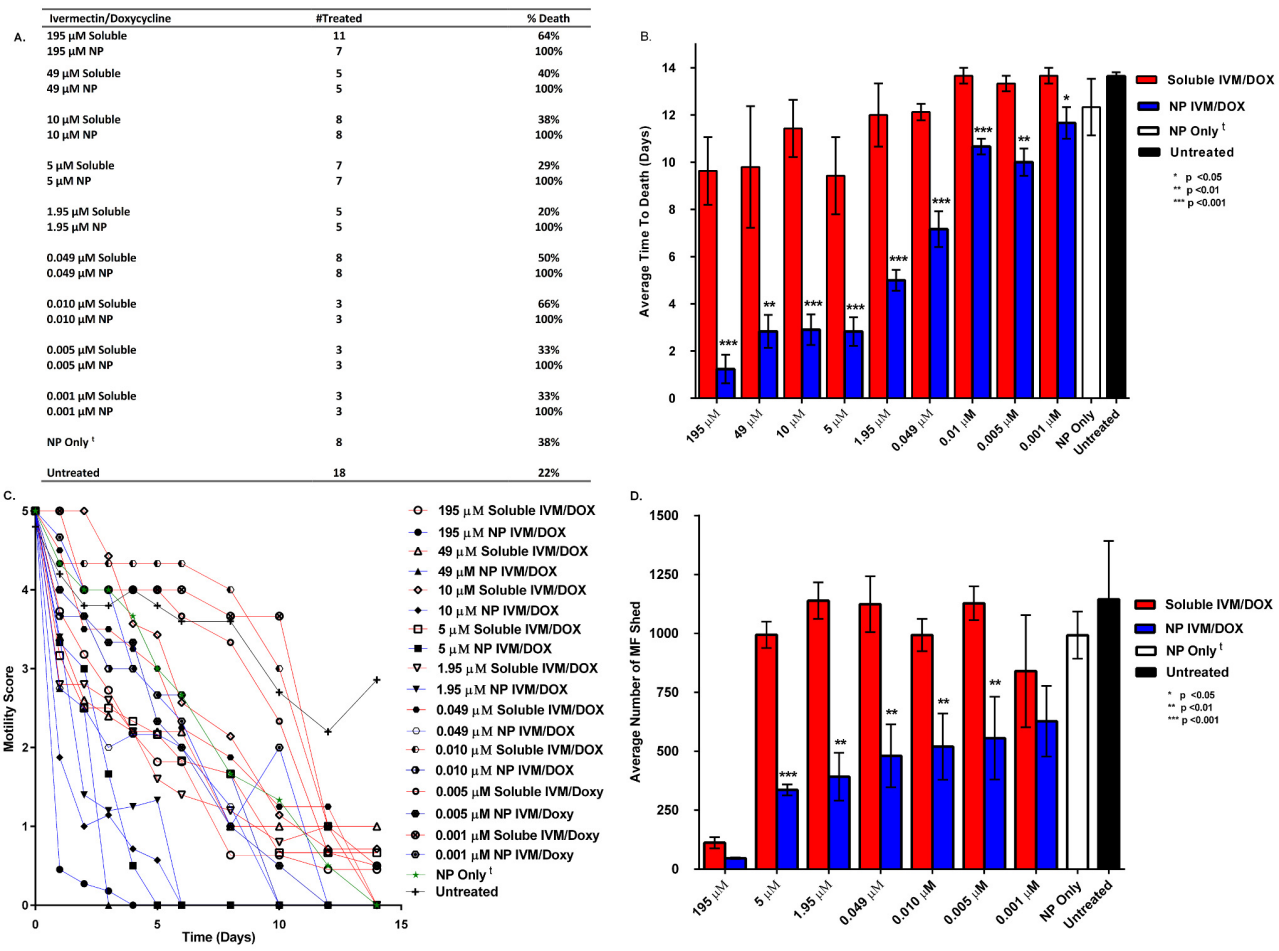
Panel A. Table outlining survival of *B*. *malayi* females after treatment with 195, 49, 10, 5, 1.95, or 0.049 μM concentrations of ivermectin (IVM) and doxycycline (DOX) delivered solubly or encapsulated into 20:80 CPTEG:CPH nanoparticles. Panel B. Average number of days to death of *B*. *malayi* females after administration of soluble or encapsulated IVM/DOX treatments and comparison to control worms. Significance was determined at p<0.05, 0.01, or 0.001 as noted using a Student’s T test. Panel C. Average motility scores of *B*. *malayi* females after IVM/DOX treatments scored using a 2X objective on a Nikon Microscope following a 0–5 scoring system, as described in the Methods. Panel D. Average number of microfilaria shed by *B*. *malayi* females after administration of soluble or encapsulated IVM/DOX treatments and comparison to control worms. The NP only group contains comparable amount of rhodamine and the total amount of particle in this group corresponds to that of the highest drug concentration of 195 μM. At 14 days, all worms treated with NP only with a motility score of 0 remained viable based on the MTT assay and recovery of motility upon transferring to fresh medium^†^. Significance was determined at p<0.05, 0.01, or 0.001 as noted using a Student’s T test.

**Fig 3:**
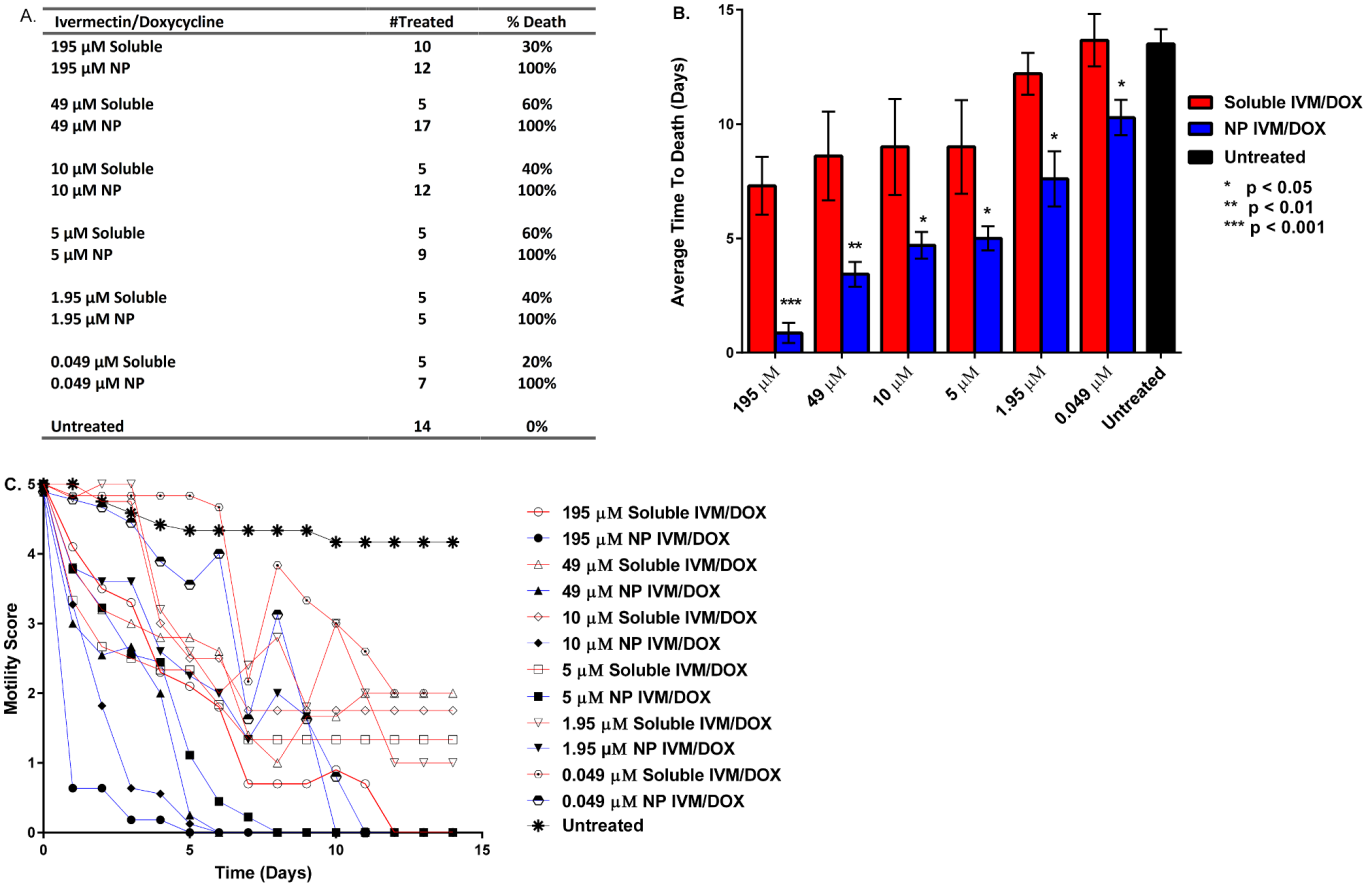
Panel A. Table outlining survival of *B*. *malayi* males after treatment with 195, 49, 10, 5, 1.95, or 0.049 μM concentrations of ivermectin (IVM) and doxycycline (DOX) delivered solubly or encapsulated into 20:80 CPTEG:CPH nanoparticles. Panel B. Average number of days to death of *B*. *malayi* males after soluble or encapsulated IVM/DOX treatments as compared to control worms. Significance was determined at p<0.05, 0.01, or 0.001 as noted using a Student’s T test. Panel C. Average motility scores of *B*. *malayi* males after IVM/DOX treatments scored using a 2X objective on a Nikon Microscope following a 0–5 scoring system, as described in the methods.

The effectiveness of the polyanhydride nanoparticle-based delivery platform was compared to that of the standard soluble treatment in terms of the overall percent survival of *B*. *malayi* over the duration of the study with a single treatment of the previously described groups (Figs 2 and 3, panel A). The effect of blank (i.e., no drug) nanoparticles containing only rhodamine was assessed with 2 mg/mL of nanoparticles, the amount corresponding to that of the highest drug concentration administered (i.e., 195 μM). Similarly, untreated controls did not contain active drugs, but were supplemented with rhodamine and DMSO corresponding to the amounts present in the highest treatment group (i.e., 195 μM drug concentration). The motility of the worms treated with the blank nanoparticles did not vary significantly from that of the untreated worms for either female or male worms (Figs 2 and 3 and S1 Fig). The results clearly demonstrate the effectiveness of the nanoparticle formulations for all the treatment groups when compared to the soluble treatment group for drug concentrations as low as 1 nM. Another parameter to quantify the difference between the effectiveness of the soluble dual ivermectin/doxycycline treatment and that of the nanoparticle-based delivery platform was the average number of days to death. We conducted a fourteen-day *in vitro* study that quantified the average days to death of female (Fig 2, panel B) and male (Fig 3, panel B) *B*. *malayi*. Random testing of viability of worms exhibiting a motility score of 0 for metabolic activity was performed using the MTT reduction assay throughout the studies and for all treatment groups and a representative of % MTT inhibition analysis is shown in S2 Fig for the 5 μM drug concentration. Inhibition of metabolic activity greater than 75% of that of untreated controls indicated that worms were non-viable and declared dead. In addition, separate worms with a motility score of 0 were subjected to reversibility testing whereby after 24 h of no motility, worms were removed from the test media, washed three times and placed in fresh media with no drugs. Recovery of movement was interpreted as the worms being temporarily paralyzed and those worms were removed from the ATD, except where indicated^†^ (Figs 2 and 3, panel A). We observed reversible paralysis only when high doses of soluble drug (49 μM—195 μM) were used in approximately 25% of all worms treated in these groups. Strikingly, reversible paralysis was not observed in any nanoparticle-treated group of any drug concentration, showing that the nanoparticle formulations effectively killed the worms. The data indicated that the nanoparticle-based delivery platform significantly (p < 0.001) lowered the overall time to death when compared directly to the soluble dual ivermectin/doxycycline treatment. Treatment of female worms with 195 μM of the soluble drugs resulted in an average time of death in excess of nine days and 63% of the worms were killed (Fig 2, panels A and B). In contrast, treating the worms with the nanoparticle-based delivery system with the same drug concentration sharply reduced the average time to death to less than 1.2 days, and more significantly, 100% of the worms were killed. We also performed dose titration studies and systematically lowered the concentration of the two drugs from 195 μM to 1 nM. Female worms treated with the 1 nM concentration of soluble ivermectin/doxycycline resulted in an average time to death that was >14 days, and more significantly, only 33% of the worms were killed at this concentration (Fig 2, panel A). In contrast, the worms treated with the nanoparticle-based delivery system containing the 1 nM drug concentration had an average time to death of <6 days, and more significantly, 100% macrofilarial death was observed upon treatment with the low concentration of ivermectin/doxycycline.

**Fig 4:**
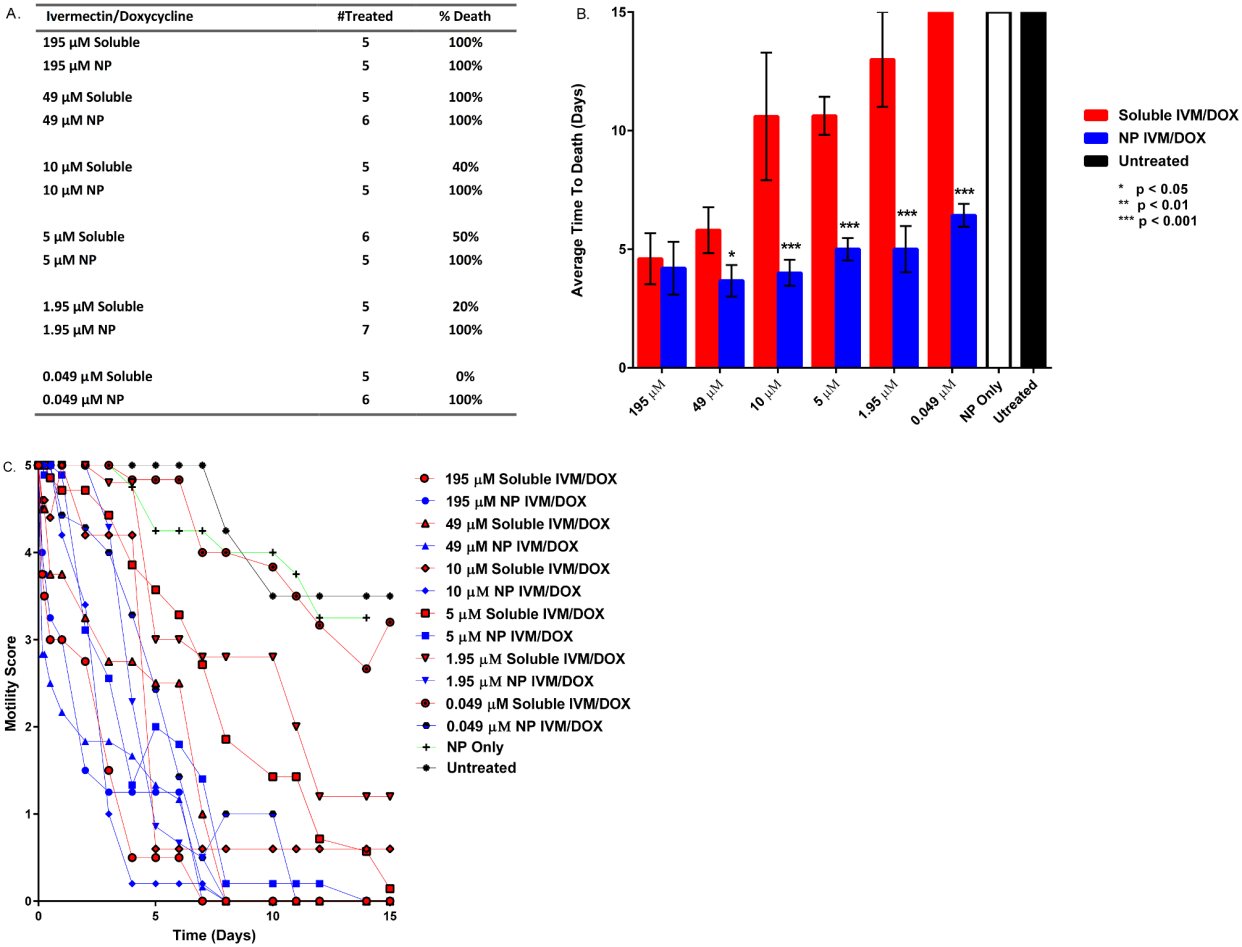
The motility of *B*. *malayi* MF treated with decreasing doses of either soluble or encapsulated ivermectin (IVM)/doxycycline (DOX) was recorded for 14 days post treatment. The recorded motility scores (Panel B) were used to calculate an average time to death for each treatment group and dose (Panel A). To calculate the average motility score for each time, dose and treatment group, triplicate wells containing a minimum of 200 MF in each well were treated as indicated. A motility score of 0 was equated with death and the average time to death was plotted along with standard error. Data presented are from one of two experiments with similar results. Significance was determined at p<0.05, 0.01, or 0.001 as noted using a Student’s T test.

To determine if nanoparticles changed the reproductive capacity of female worms the shedding of MF was observed for fourteen days (Fig 2, panel D). Both the soluble ivermectin/doxycycline and the nanoparticle-based delivery groups significantly reduced the overall microfilariae shed at the high drug concentration of 195 μM. But as the concentration of ivermectin/doxycycline was systematically reduced to 5 nM, significant differences (p<0.01) were observed in the MF shed by the worms treated with the soluble ivermectin/doxycycline groups compared to the worms that were treated with the nanoparticle-based delivery system, with the nanoparticles being more effective at lower drug concentrations. The effect of the various drug treatments on the motility of *B*. *malayi* MF was observed for 14 days (Fig 4). Similar to the effects observed for adults, the average time to death as well as the motility of the MF treated with the soluble drug was significantly (p < 0.001) higher in comparison to that of the MF that were treated with the nanoparticle-based delivery system, especially at low drug concentrations.

For the *B*. *malayi* males (Fig 3, panels A and B) treatment with drug-loaded 20:80 CPTEG:CPH nanoparticles showed a significant difference (p<0.05) in terms of survival when compared to the survival of worms treated with an equivalent soluble dose of ivermectin/doxycycline. In terms of average time to death, male worms treated with a 195 μM concentration of soluble ivermectin/doxycycline died after seven days and more significantly, only 30% of them were killed at this dose (Fig 3, panel A). In sharp contrast, all the male worms treated with the nanoparticle-based delivery system containing the same dose of ivermectin/doxycycline died in less than one day. When the treatment concentration was reduced to 0.049 μM, the average time to death of worms treated with soluble ivermectin/doxycycline was >14 days with a death rate of only 20%. On the other hand, all the male worms treated with the nanoparticle-based delivery system containing the 0.049 μM concentration of ivermectin/doxycycline died within ten days.

In conjunction with the average time to death assay we also monitored the motility over time to characterize the overall effectiveness of the nanoparticle treatments (panel C of Figs 2 and 3). With both male and female *B*. *malayi* worms, an exponential reduction in overall motility was observed in the worms administered the nanoparticle-based delivery treatments in comparison to that of the worms that received the soluble drug treatment.

### Polyanhydride nanoparticle-based treatment effectively penetrated the worm cuticle

**Fig 5:**
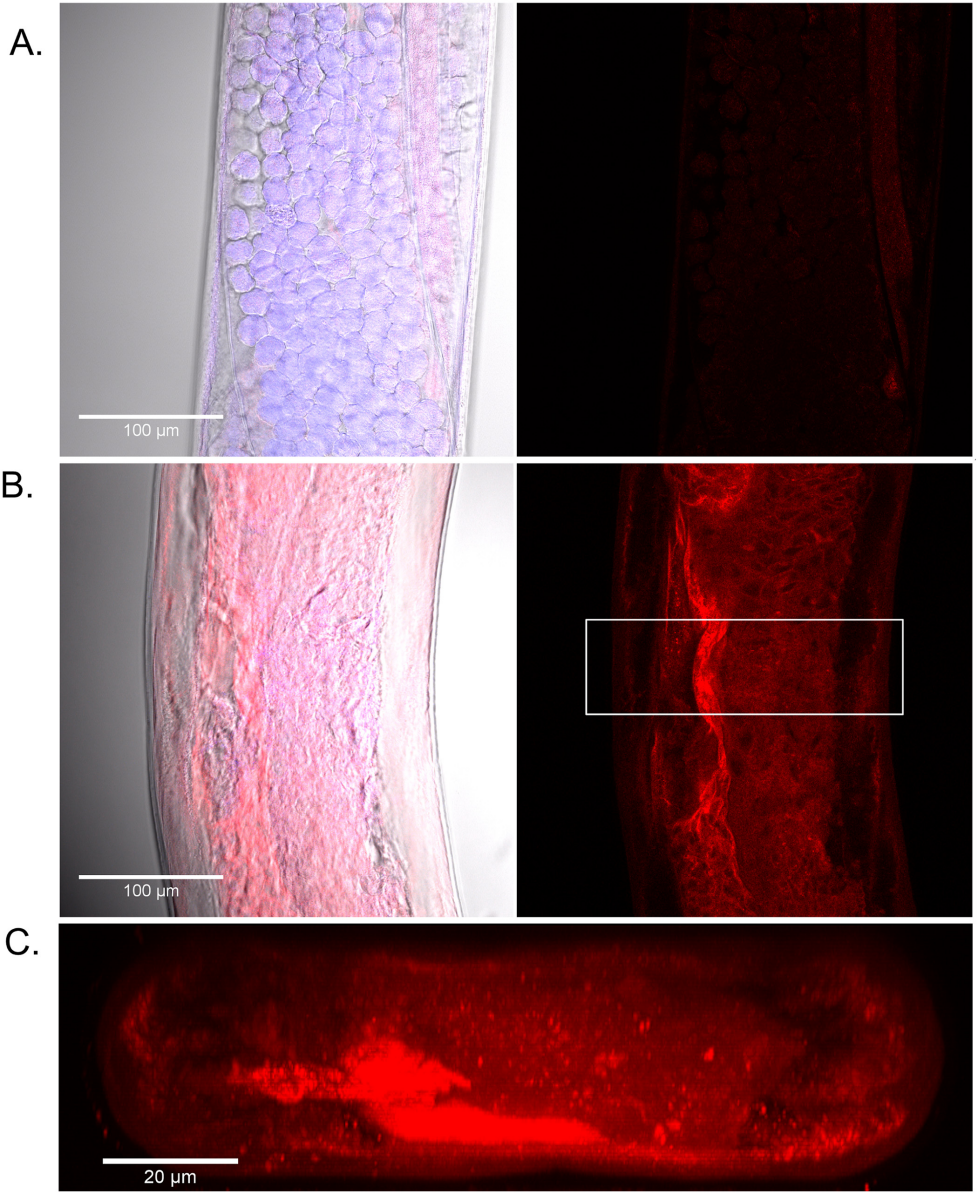
Confocal microscopy of female *B*. *malayi* with nanoparticles. Worms were incubated for 96 h with either soluble controls (panel A) or 20:80 CPTEG:CPH nanoparticles containing ivermectin (IVM), doxycycline (DOX), and rhodamine B (panel B), fixed and imaged by LSCM. Controls contained 195 μM each of IVM and DOX, and 3.9 μM rhodamine B, while the nanoparticles contained 5 μM of each drug and 0.1 μM of rhodamine B. Left panels are DNA (blue), rhodamine (red) and bright field image overlays and right panels are the respective individual rhodamine images collected using identical image acquisition settings. Inset box within the nanoparticle-treated worm outlines the area selected for side view rendering (C). Representative images shown demonstrate the accumulation of nanoparticles within tissues throughout the worm (B) as compared to the higher amount of soluble rhodamine that was not detected within the body of the worms.

To visualize the interactions of polyanhydride nanoparticles with *B*. *malayi* female worms, animals were treated with equivalent amounts of a cocktail containing rhodamine red dye with ivermectin/doxycycline either in soluble form or encapsulated within the 20:80 CPTEG:CPH nanoparticles. At regular intervals worms were washed three times in PBS to remove surface-bound nanoparticles, fixed, and then imaged using confocal microscopy to localize nanoparticles within tissues. Detection of focal, intense rhodamine staining is consistent with size and staining patterns of intact nanoparticles. In comparison, diffuse red staining is indicative of free rhodamine. Worms treated with 195 μM of the soluble drug cocktail did not accumulate rhodamine within tissues over a period of 96 hours (Fig 5, panel A). Over this same time interval, the much lower dose of 5 μM of ivermectin/doxycycline encapsulated within the nanoparticles that also contained rhodamine, extensively labeled the interior structures of female worms (Fig 5, panel B). Diffuse red staining reveals the rapid distribution of the released dye throughout the body of the worm and focal, intact 20:80 CPTEG:CPH nanoparticles are easily discernable deep within worm tissues by confocal microscopy (Fig 5, panels B and C). Three-dimensional reconstruction of confocal Z-stacked images revealed the penetration of nanoparticles and release of rhodamine throughout the inner tissues of the worm (Fig 5, panel C). Similar rhodamine and nanoparticle staining patterns were evident throughout the length of the treated worms at all concentrations tested. Internal staining of worms treated five hours post treatment with 195 μM of the soluble drug cocktail were identical to the rhodamine intensity of worms treated with 5 μM of the drug cocktail encapsulated within the nanoparticles for 96 hours. These observations offer insights into why the nanoparticle-based delivery treatments of *B*. *malayi* are highly effective, especially at low doses.

## Discussion

The World Health Organization global program to eliminate LF recommends annual dosing of albendazole with either ivermectin or diethylcarbamazine to be continued for at least five years. These drugs are potent anthelmintics that kill and reduce shedding of MF. However, they exhibit little direct activity against the adult filarial worms [41]. Discovery of the endosymbiotic bacteria *Wolbachia* and the critical role they play in the biology and viability of the adult worm revealed a potential target that could be exploited to improve therapies for RB and LF [19,21,42]. The addition of anti-bacterials (such as doxycycline or tetracycline) to the treatment regimen can reduce parasite embryogenesis [20,43], improve macrofilaricidal activity, and reduce hydrocele pathology in the host [44,45]. To achieve maximal benefit, daily dosing of 200 mg for 3–5 weeks of antibacterial drug is required and can be a difficult barrier to overcome for Mass Drug Administration (MDA) programs [15,27]. In this work, we report that the use of a polyanhydride nanoparticle-based carrier effectively reduced the amount of drug necessary for macrofilarial death by up to 4,000-fold compared to physiologically relevant doses.

The unique chemistry of the polyanhydride nanoparticles helps address many of the challenges associated with mass drug administration against lymphatic filariasis. The surface erosion profile of the polyanhydride nanoparticles sustains the slow release of drug at different kinetics for both drugs from the same particle as shown in Fig 1. The initial burst of the doxycycline released from the 20:80 CPTEG:CPH nanoparticles is faster than that observed for ivermectin. Previous work has shown that this copolymer composition contains weakly segregated CPTEG- and CPH-rich domains [46]. This may indicate a preferential partitioning of the hydrophobic ivermectin to the slowly degrading CPH-rich domains of the copolymer, leading to the slow release profile observed [35,47]. In contrast, the less hydrophobic doxycycline is released faster, suggesting partitioning into the faster eroding CPTEG-rich domains. This controlled release profile of the drugs from the polyanhydride nanoparticles may explain the enhanced efficacy observed with the nano-carrier treatments. The payload is protected from an aqueous environment for longer periods of time, which may prevent degradation of the drugs, leading to the reduction in required dose observed in this work. Additionally, based on the confocal microscopy studies described in Fig 5, it appears that these nanoparticles effectively interact with the worm cuticle and behave like a Trojan horse in terms of transporting the drug cocktail into the worm, bypassing excretory pumps. Together, the encapsulation and subsequent controlled release of the drugs at high concentrations both internally to the worm and localized to the cuticle may account for the enhanced killing observed in Figs 2 and 3.

Reducing the effective amount of drug required would have several benefits on improving therapeutic treatment of filarial diseases. We predict that increasing drug effectiveness would likely reduce unwanted side effects by reducing amount of drug needed and contribute to improving patient compliance [43,48,49]. As discussed above, we hypothesize that encapsulation and subsequent controlled release of the drug and the ability of the polyanhydride nanoparticle carriers to localize to the worm cuticle surface, and even be internalized by the worms, lead to a higher microenvironment concentration of the drugs. In contrast, the soluble drug cocktail is a liquid, and so to create the same local concentration internally to the worm and at the cuticle surface, more drug must be administered initially. This is supported by the confocal microscopy data in Fig 5, which show that the polyanhydride nanoparticles readily localize to the worm, increasing the concentration of the internalized dye as compared to the soluble control. *In vivo*, while the soluble drug will be rapidly cleared from the body, the nanoparticle-encapsulated drug will persist in different tissues for periods up to 28 days, as shown by our previous studies [50,51]. Another difficulty in treating filarial infections is that individuals are continually being exposed to new parasites even after successful completion of antiparasitic regimen [52]. It was reported by Walker *et al*. that while standard doxycycline therapy was able to reduce the majority of macrofilaria in human subjects, reinfection with drug-naïve parasites occurred [19]. In this scenario, we would predict that the delayed release capacity of the nanoparticles would be able to extend the effective range beyond the time frame afforded by standard dosing.

The polyanhydride nanoparticle drug delivery platform decreased the average time to death of *B*. *malayi* females and facilitated a rapid decrease in the motility of treated worms as shown in Fig 2, even at nanomolar concentrations of the drugs. Similar trends were also observed in the *B*. *malayi* males to a slightly lesser extent, possibly reflecting the comparatively lower number of *Wolbachia* in male compared to female macrofilariae (Fig 3). Rapid killing would likely contribute to the dramatic reduction in shedding by female worms at low nanoparticle concentrations (Fig 2, panel D). Effectiveness at the lower concentrations may prove to be more beneficial as rapid killing of MF is known to induce severe side-effects within the host [53]. It is also likely that the nanoparticles increased the rate of delivery of ivermectin and rapidly paralyzed the muscles within the body of the worm and the vagina, which are needed to expel the MF. A similar *in vitro* observation was made by Tompkins *et al*. with *Brugia* treated with either moxidectin or ivermectin [41].

Finally, the confocal microscopy data in Fig 5 provides an initial explanation of the strong interactions between polyanhydride nanoparticles and the cuticle surface, which is hypothesized to lead to the efficacy profile of this nano-carrier platform. Tracking the fluorescent rhodamine B co-loaded into the nanoparticles demonstrated a greater increased influx of payload into the worm compared to drug and dye delivered solubly. As previously stated, in addition to providing sustained release of drug, with implications for dose sparing and patient compliance, the drug-loaded polyanhydride nanoparticles demonstrated enhanced internalization by the *B*. *malayi* worms. One of the benefits of this type of Trojan horse behavior of the nanoparticles is the ability to create microenvironments with high drug concentrations, in contrast to soluble drug cocktail treatments, which diffuse in an aqueous solution [54]. To achieve the benefit of an increased local drug concentration, the nanoparticles must interact with the target cell, or, in this case, the *B*. *malayi* adult worm or microfilaria. The data in Fig 5 clearly showed that the nanoparticles are interacting with the worms in a way not previously described, improving the rapid delivery of drugs. We speculate that the nanoparticles undergo facilitated diffusion into the worm, by crossing the cuticle, penetrating the hypodermis membrane barriers and accessing deeper tissues within the worm [55,56]. Preliminary assessment of treated worms by dissection corroborates cuticle penetration because far fewer nanoparticles were observed in the gut and pharynx of worms, while they did accumulate in deeper tissues including the hypodermis. More detailed studies are underway in our laboratories to further delineate the mechanisms of nanoparticle transport across the cuticle of the worms.

Resistance to anthelmintic drugs is on the rise due in part to the over-expression of ABC-transporters that act as multi-drug resistant (MDR) efflux pumps in many parasites, including *Onchocerca* and *Brugia* [57]. Our success with delivery of drug cocktails within nanoparticles needs to be analyzed in the context of previous work, wherein the approach of delivering drug combinations was shown to reduce the development of resistance in parasites [18]. However, adoption of cocktail therapy into MDA programs remains problematic due to the need to maintain high levels of doxycycline [18]. We posit that encapsulating low doses of these drugs into the Trojan horse polyanhydride nanoparticles address the difficulties in maintaining prolonged delivery of antiparasitic drugs. The activity of the efflux pumps would normally reduce effective drug concentrations within the worm by preventing absorption through the esophagus as well as reducing intracellular concentration within cells. We speculate based on our results that the efficient and rapid direct penetration of the polyanhydride nanoparticles through the surface of the worm would bypass poor esophageal absorption. We hypothesize that accessing the worm through this unique mechanism would be a selective pressure that would be difficult for the parasite to overcome and provide a paradigm-changing technology to combat filarial disease.

### Conclusions

Filarial diseases represent a significant social and economic burden in areas that are endemic with the filarial endoparasite *B*. *malayi*, and its symbiotic bacteria *Wolbachia*. We report the use of a polyanhydride nanoparticle-based drug delivery platform for the co-delivery of the antiparasitic drug, ivermectin, to reduce macro and microfilarial burden and the antimicrobial, doxycycline, to eliminate the symbiotic *Wolbachia*. The co-delivery of doxycycline and ivermectin in the context of polyanhydride nanoparticles effectively killed adult female and male *B*. *malayi* filarial worms with up to a 4,000-fold reduction in the amount of drug used. Further, the time to death of the macrofilaria was significantly reduced when the anti-filarial drug cocktail was delivered in the context of the polyanhydride nanoparticles. Confocal microscopy experiments suggest that the polyanhydride nanoparticles behave like a Trojan horse and penetrate the outer membrane of the worm cuticle. The nanoparticles effectively deliver the drugs at high enough microenvironment concentrations to vital areas within the worm, thus significantly enhancing their effectiveness at killing the worms. These findings may have significant consequences for the reduction in the amount of drug and the length of treatment required for filarial infections and provide a paradigm-changing technology to combat filarial disease.

## Supporting Information

S1 Fig Synergism of ivermectin (IVM) and doxycycline (DOX) in reducing motility of adult *B*. *malayi* females following encapsulation of drugs into nanoparticles. Average time to death of three worms treated in separate wells is shown in Panel B. Average motility scores of *B*. *malayi* females (Panel C) after treatments where visually scored with the 0–5 scoring system, as described in the methods. Panel D Indicates the use of the “Worminator”, a computer motion screening program utilized for motility scoring in *B*. *malayi* (Marcellino C, Gut J, Lim KC, Singh R, McKerrow J, & Sakanari J (2012). WormAssay: a novel computer application for whole-plate motion-based screening of macroscopic parasites. PLoS Negl Trop Dis 6(1): e1494). The percent dead were worms with a motility score of 0 (Panel A) and confirmed by MTT to show inhibition greater than 80% of untreated control.
(TIF)

S2 Fig Enzymatic reduction of MTT reagent by adult *B*. *malayi* females following incubation with various treatments for 9 days. Motility of worms tested by the MTT assay is shown in S1 Fig. Inhibition was calculated using optical density values of reduction of MTT reagent for untreated worms to represent maximum metabolic activity (Panel A). Changes in reduction of MTT recorded values for soluble and nanoparticle-based drug treatment groups as compared to untreated worms were calculated as percent inhibition (Panel B). Three individually treated worms were used for each treatment group and individual MTT percent inhibition was used to calculate the average and standard error. Significance difference of *p*<0.01 was calculated between the soluble IVM/DOX and the nanoparticle-encapsulated IVM/DOX. Inhibition greater than 75% was interpreted as loss in viability.
(TIF)
